# Evolution and developmental expression of the sodium–iodide symporter ( *NIS* , *slc5a5*) gene family: Implications for perchlorate toxicology

**DOI:** 10.1111/eva.13424

**Published:** 2022-07-07

**Authors:** Ann M. Petersen, Clayton M. Small, Yi‐Lin Yan, Catherine Wilson, Peter Batzel, Ruth A. Bremiller, C. Loren Buck, Frank A. von Hippel, William A. Cresko, John H. Postlethwait

**Affiliations:** ^1^ Department of Biology, Institute of Ecology and Evolution University of Oregon Eugene Oregon USA; ^2^ J.J. Howard Marine Lab, Northeast Fisheries Science Center National Oceanographic and Atmospheric Administration Sandy Hook New Jersey USA; ^3^ Department of Biology, Institute of Neuroscience University of Oregon Eugene Oregon USA; ^4^ Department of Biological Sciences Northern Arizona University Flagstaff Arizona USA; ^5^ Department of Community, Environment & Policy, Mel & Enid Zuckerman College of Public Health University of Arizona Tucson Arizona USA

**Keywords:** gene evolution, NIS, perchlorate, *slc5a5*, stickleback, thyroid

## Abstract

The vertebrate sodium–iodide symporter (NIS or SLC5A5) transports iodide into the thyroid follicular cells that synthesize thyroid hormone. The SLC5A protein family includes transporters of vitamins, minerals, and nutrients. Disruption of SLC5A5 function by perchlorate, a pervasive environmental contaminant, leads to human pathologies, especially hypothyroidism. Perchlorate also disrupts the sexual development of model animals, including threespine stickleback (*Gasterosteus aculeatus*) and zebrafish (*Danio rerio*), but the mechanism of action is unknown. To test the hypothesis that SLC5A5 paralogs are expressed in tissues necessary for the development of reproductive organs, and therefore are plausible candidates to mediate the effects of perchlorate on sexual development, we first investigated the evolutionary history of *Slc5a* paralogs to better understand potential functional trajectories of the gene family. We identified two clades of *slc5a* paralogs with respect to an outgroup of sodium/choline cotransporters (*slc5a7*); these clades are the NIS clade of sodium/iodide and lactate cotransporters (*slc5a5*, *slc5a6*, *slc5a8*, *slc5a8*, and *slc5a12*) and the SGLT clade of sodium/glucose cotransporters (*slc5a1*, *slc5a2*, *slc5a3*, *slc5a4*, *slc5a10*, and *slc5a11*). We also characterized expression patterns of *slc5a* genes during development. Stickleback embryos and early larvae expressed NIS clade genes in connective tissue, cartilage, teeth, and thyroid. Stickleback males and females expressed *slc5a5* and its paralogs in gonads. Single‐cell transcriptomics (scRNA‐seq) on zebrafish sex‐genotyped gonads revealed that NIS clade‐expressing cells included germ cells (*slc5a5*, *slc5a6a*, and *slc5a6b*) and gonadal soma cells (*slc5a8l*). These results are consistent with the hypothesis that perchlorate exerts its effects on sexual development by interacting with *slc5a5* or its paralogs in reproductive tissues. These findings show novel expression domains of *slc5* genes in stickleback and zebrafish, which suggest similar functions across vertebrates including humans, and provide candidates to mediate the effects of perchlorate on sexual development.

## 1 INTRODUCTION

Transport of molecules across membranes is essential for cells to maintain homeostasis. Active solute cotransporters move low molecular weight solutes across cell membranes. Solute carrier (*SLC*) genes have evolved into at least 52 families comprising about 400 genes in the human genome (Hediger et al., 2013; Wright & Turk, 2004), underscoring their physiological importance in many different cellular processes. Mutations in human *SLC* genes have been linked to a wide array of diseases, including hypothyroidism, glycogen storage disease, and deafness (Fujiwara et al., 1997; Hediger et al., 2013). Furthermore, some *SLC* genes are drug targets for pharmaceutical therapies, making these transporters of biomedical interest (Hediger et al., 2013; Wright & Turk, 2004).

The SLC5A protein family includes transporters of vitamins, minerals, and nutrients (Wright et al., 2011). SLC5A genes have been described in basally diverging lobe‐finned vertebrates as well as derived terrestrial vertebrates (including coelacanth and frogs; Eid et al., 2002), ray‐finned fishes (Subramaniam et al., 2019), protostomes (e.g., insects; Caccia et al., 2007), and crustaceans (Obi et al., 2011). The widespread phylogenetic distribution of SLC5A proteins provides an opportunity to investigate their conserved, biomedically important functions, including dysregulation during development in nonmammalian vertebrates. The vertebrate SLC5A family includes at least 12 members (SLC5A1–SLC5A12), mutations which are associated with human pathologies, including glucose–galactose malabsorption (SLC5A1, Xin & Wang, 2011), renal glucosuria (SLC5A2, van den Heuvel et al., 2002), thyroid dyshormonogenesis (SLC5A5, Kosugi et al., 1998), infantile‐onset biotin‐responsive neurodegeneration (SLC5A6, Byrne et al., 2019), and distal hereditary motor neuronopathy VIIA (SLC5A7, Barwick et al., 2012).

A subset of *SLC5A* family genes encode sodium–glucose transporters (SGLT) involved in glucose homeostasis and uptake in the mammalian intestine (Wright et al., 2011). Less is known about SLC5A family members that have a substrate preference for solutes other than glucose, including transporters for iodide/sodium (SLC5A5), vitamins (SLC5A6), iodide and lactate (SLC5A8), and glucose/sodium (SLC5A12). SLC5A8 transports iodide by a passive mechanism (Rodriguez et al., 2002) and monocarboxylates and short‐chain fatty acids by a sodium‐coupled mechanism (Gopal et al., 2004). The sodium–iodide symporter (NIS, alias SLC5A5) cotransports sodium (Na^+^) and iodide (I^−^) into cells across the basolateral plasma membrane (Ravera et al., 2017). Humans express *Slc5a5* mainly in stomach, salivary glands, and especially in thyroid follicular cells, where it supplies iodide for thyroid hormone production. *Slc5a5* in humans is also expressed in breast, colon, bladder, kidney, pancreas, prostate, and ovary, although its function in many of these organs is unknown (Wright et al., 2011).

SLC5A5 activity is sensitive to the small molecule perchlorate, a common aquatic pollutant that is used as an oxidizer for consumer and industrial applications such as propellants, fireworks, airbags, matches, and flares (Kendall & Smith, 2006; Urbansky et al., 2001). Perchlorate contaminates drinking water in many places and many commonly consumed food products (Murray et al., 2008). It is often present in human breast milk and urine (De Groef et al., 2006; Urbansky, 2002). Perchlorate and other compounds that are electrochemically similar to iodide, including thiocyanate, nitrate, tetraflouraborate, and at least seven others (Eskandari et al., 1997), competitively inhibit SLC5A5 in several species (Bradford et al., 2005). Low doses of perchlorate exposure up‐regulate *slc5a5* gene expression at the level of RNA in mammals (McDougal et al., 2011). In addition, perchlorate levels that do not affect circulating thyroid hormone concentrations nevertheless compromise the iodide concentrating function of thyroid follicular cells (Lawrence et al., 2000). Understanding the evolutionary history and sequence similarities of this gene family, as well as their temporal and spatial developmental expression patterns, will provide insight into the mechanisms by which the endocrine disruptor perchlorate causes disease in vertebrates. Use of comparative molecular and sequence analysis data in an evolutionary framework applied to toxicology is an emerging and important new tool for toxicogenomics (Oziolor et al., 2017).

Threespine stickleback (*Gasterosteus aculeatus*) is an important natural model for investigations of evolution, physiology, and environmental toxicants (Bell, 1994; Cresko et al., 2004; Gravenmier et al., 2005) and is therefore appropriate to use in this study. Stickleback reproductive development has been well described (Baker et al., 2008). Male and female stickleback exhibit identifiable physiological markers of normal reproductive development, such as the androgen‐induced production of nest‐building glue (spiggin) by the kidney in males (Jakobsson et al., 1999), and the production of vitellogenin by the liver and mature eggs in the ovary of females (Katsiadaki et al., 2007). In addition, stickleback has a well‐annotated genome (Jones et al., 2012), as well as an XY system of sex determination with sex‐linked markers making genetic sex identifiable by PCR independent of phenotypic sex (Griffiths et al., 2000; Ross et al., 2009). A potentially crucial time period in sex determination and reproductive development of stickleback occurs between 15 and 18 days postfertilization (dpf) because primary germ cell (PGC) numbers in the developing gonad of genotypic females first become higher than those in genotypic males during this developmental window, a trend that continues throughout the rest of gonadogenesis (Lewis et al., 2008). As PGC number increases in females, apoptosis increases in somatic cells of the developing gonad (Lewis et al., 2008; Petersen et al., 2016). We previously found that perchlorate exposure alters sexual development in threespine stickleback (Bernardt & von Hippel, 2008; Bernhardt et al., 2006; Furin, von Hippel, Cresko, et al., 2015b), disrupts androgen production (Petersen et al., 2015), and decreases primordial germ cell number (Petersen et al., 2016).

We hypothesize that perchlorate may exert its effects on stickleback reproduction by mechanisms independent of *slc5a5* activity in the thyroid by acting on Slc5a5 or its paralogs in extra‐thyroidal cells, likely in the gonads (the ”paralog/gonad hypothesis”) and possibly during this crucial time window of PGC mitosis and apoptosis. Our hypothesis predicts that *slc5a5* and/or its paralogs should be expressed in the developing gonad and provide a target sensitive to perchlorate leading to reproductive pathologies, including masculinization associated with exposure to perchlorate and its inhibition of *slc5a5* function (Bernhardt et al., 2011). Given the substrate versatility of several members of the *slc5a* gene family (de Carvalho & Quick, 2011; Eskandari et al., 1997), we hypothesize that perchlorate affects the development of reproductive organs by disrupting the function not only of SLC5A5 in the thyroid but also by acting on SLC5A5 and/or SLC5A5‐related proteins expressed in nonthyroidal tissues, such as the gonad. The paralog/gonad hypothesis predicts that *slc5a5* and possibly one or more of its closest paralogs will be expressed in organs other than the thyroid.

Evaluating this prediction in teleost fishes such as stickleback first requires the identification of orthologs and the closest paralogs of *slc5a5* in teleost genomes. This work is sometimes difficult due to: (1) the teleost genome duplication (Amores et al., 1998; Braasch et al., 2016; Jaillon et al., 2004; Postlethwait et al., 1998), (2) lineage‐specific loss of ohnologs, which are paralogs derived from genome duplication (Braasch et al., 2016), and (3) the evolution of novel tissue‐ and time‐specific gene expression patterns after genome duplication (Force et al., 1999). The work described here helps to fill these knowledge gaps and to evaluate the potential for SLC5A family members to mediate nonthyroidal effects of perchlorate exposure on development.

Because the paralog/gonad hypothesis predicts that *slc5a* paralogs should be expressed in organs related to reproduction during the sensitive period for stickleback sex determination, we investigated the spatial and temporal expression patterns of *slc5a5* and its paralogs using in situ hybridization. To identify stickleback *slc5a* paralogs, we performed phylogenetic and conserved synteny analyses to find orthologs and lineage‐specific duplicates of a single ortholog (co‐orthologs) of human genes most closely related to *SLC5A5*. We then investigated their gene expression patterns in the thyroid and gonad during stickleback development, and we characterized their expression in male and female zebrafish (*Danio rerio*) gonads using single‐cell RNA‐seq. Mapping the spatial and temporal expression of *slc5a5* and its paralogs allows us to better understand conserved and derived functions of this gene family in teleosts specifically, and in vertebrates generally. Future manipulative studies should test whether perchlorate also disrupts the function of *slc5a5* paralogs, thereby providing a possible mechanism to explain how some chemicals that affect solute transport may disrupt reproductive development.

## 2 MATERIALS AND METHODS

### 2.1 Animal care

Laboratory‐bred threespine stickleback originally collected from Rabbit Slough (N 61.5595, W 149.2583) near Cook Inlet, Alaska (Cresko et al., 2004), and reared in the laboratory for at least eight generations were used for all experiments. Beginning at hatching at about 6 dpf, fry were fed with *Artemia* (Inve Aquaculture, Inc.). Fish and fry of all ages were maintained in 6 ppt Instant Ocean (Aquarium Systems) added to reverse osmosis water at a constant temperature of 20°C. Adult fish were maintained on a long daylight cycle of 20 h of light and 4 h dark to simulate summer conditions in their natural habitat; juvenile fish were on a short daylight cycle of 10 h light and 14 h dark. Animal care, experimental methods, and euthanasia were approved by our University's Institutional Animal Care and Use Committee (protocol #10–16).

### 2.2 Paralog identification and phylogenetic reconstruction

We obtained SLC5A nucleotide and amino acid sequences from the reference genomes of threespine stickleback, zebrafish, spotted gar (*Lepisosteus oculatus*), coelacanth (*Latimeria chalumnae*), mouse (*Mus musculus*), and human (*Homo sapiens*) using annotated gene families in Ensembl (Zerbino et al., 2018; Figure S2). We aligned protein sequences with MAFFT v7 (Katoh & Standley, 2013) using the options ‐‐localpair and ‐‐maxiterate 1000. We then adjusted alignments manually by removing poorly aligned residues. Specifically, we removed unalignable N and C termini, and two poorly aligned internal blocks that were 33 and 84 amino acid alignment positions in length. The trimmed alignment consisted of 88 sequences, each with 471 aligned positions. We used the PhyML 3.0 web server (Guindon et al., 2010) for Akaike information criterion (AIC) model selection and maximum‐likelihood (ML) phylogenetic inference (Guindon & Gascuel, 2003). The LG + G + F model was selected as the best fit for the alignment (AIC = 57974.39). We performed two separate evaluations of ML tree clade support: PhyML's fast SH‐like approximate likelihood ratio test (aLRT) and 100 bootstrap replicates. Phylogenetic tree visualizations were rooted assuming the SLC5A7 clade as an outgroup, an assumption inferred from its likely evolutionary distance in Ensembl gene trees and a long terminal branch leading to SLC5A7 in an unrooted topology of human SLC5A proteins (Wright, 2013).

### 2.3 Synteny analysis and nomenclature conventions

Nomenclature rules for vertebrate genes and proteins follow accepted species‐specific conventions, for example: (format below is common name (species), gene, protein, and [URL]): zebrafish (*Danio rerio*), *slc5a5*, Slc5a5, [https://wiki.zfin.org/display/general/ZFIN+Zebrafish+Nomenclature+Guidelines]; mouse (*Mus musculus*), *Slc5a5*, Slc5a5, [http://www.informatics.jax.org/mgihome/nomen/gene.shtml]; human (*Homo sapiens*), *SLC5A5*, SLC5A5 [http://www.genenames.org/]; frog (*Xenopus tropicalis*), *slc5a5*, Slc5a5 [http://www.xenbase.org/gene/static/geneNomenclature.jsp]; chicken (*Gallus gallus*) and turkey (*Meleagris gallopavo*), *SLC5A5*, SLC5A5 [http://birdgenenames.org/cgnc/guidelines]; and fruit fly (*Drosophila melanogaster*), *CG10444*, CG10444 [http://flybase.org/static_pages/docs/nomenclature/nomenclature3.html]. For species that lack formalized gene and protein nomenclature conventions, we apply zebrafish nomenclature conventions to threespine stickleback, spotted gar, and coelacanth, and we apply mouse conventions to nonhuman mammals in general. Conserved synteny and dot‐plot analyses were performed using the Synteny Database http://syntenydb.uoregon.edu/synteny_db/ (Catchen et al., 2009).

### 2.4 Histological analysis

Fish were euthanized in buffered MS‐222 (Sigma‐Aldrich) and immediately fixed in either Bouin's solution or 4% Paraformaldehyde (PFA) and kept at 4°C for 48 h. After fixation, Bouin‐fixed fish were stored in 70% ethanol at 4°C. Fish fixed in PFA were stored in 100% methanol at 4°C until sectioning. Bouin‐fixed fish were paraffin embedded, sectioned at 16 μm, and stained with hematoxylin and eosin (H&E). PFA‐fixed fish were cryosectioned at 8 μm and stored at −20°C until analysis by in situ hybridization or immunohistochemistry. After staining, sections were covered with Permount (FisherChemicals), coverslipped, and left to dry overnight before analysis.

### 2.5 In situ hybridization

In situ hybridization experiments on cryosections were performed following the protocol described by Strähle et al. (1994) and Rodríguez‐Marí et al. (2005). Primers for the four *slc5a5*‐clade genes most closely related to *slc5a5* in stickleback (*slc5a5; slc5a6a*, *slc5a6b*, *slc5a8a*, and *slc5a8b*) were designed using Geneious (Biomatters) based on sequences available in Ensembl. To clone and prepare in situ probes, we used primers with sequences: gacslc5a5 F TTTAGTGTCGACCCACGTCA and gacslc5a5 R AGAGCCACAGAAGCCCACTA; gacslc5a8 F GTACCGCTACGGAGCCAATA and gacslc5a8RAGGATGCCACCTTGTACGAC; gacslc5a6b TATGCTGGAGGGTTTTCCTG and gacslc5a6b R CCCTGGTGTCACATCCTCTT; and gacslc5a6a F TCCAGCGCCTACGAGTATCT and gacslc5a6a R TAGCGAGCAAACATGACCAG. Probes for *slc5a* family genes were diluted in denatured hybridization buffer and drops of the solution were added to the sections in a humid chamber. Slides were coverslipped and hybridized overnight at 68°C and then washed in formamide washing solution at 68°C, and then with malic acid buffer. Horizontal cryosections of at least two fish from each treatment were thawed and brought to room temperature for at least 30 min before exposure to air. Probes for *slc5a* family genes were diluted in denatured hybridization buffer and drops of the solution were added to the sections in a humid chamber. Slides were coverslipped and hybridized overnight at 70°C. The following day, slides were washed in 1X saline sodium citrate solution/ 50% formamide/ 0.1% Tween 20 at 70°C and then transferred to a Coplin jar of MABT (NaCl 750 mM, Maleic acid 500 mM, and Tween 20 0.5%). Slides were drained, treated with a blocking solution of 8% BSA, and incubated for 1 h at room temperature. Slides were drained, topped with 1:5000 antidigoxigenin antibody solution, and incubated overnight at room temperature. The following day, alkaline phosphate staining buffer in polyvinyl alcohol was added to the slides. Slides were incubated overnight in 4‐nitro blue tetrazolium chloride solution (NBT) and 5‐bromo, 4‐chloro, 3‐indolylphosphate (BCIP) in polyvinyl alcohol solution at 37°C. Staining was stopped by washing in 2XPBS +0.1% Tween. Slides were then dehydrated stepwise in an ethanol series, cleared in xylene, and coverslipped in Permount.

### 2.6 Thyroglobulin antibody staining

Horizontal cryosections of at least two fish from each treatment were thawed and brought to room temperature for at least 30 min before exposing to air. Slides were washed with 1X sterile PBS with 0.1% Tween and placed in a sealed humid container. Slides were not allowed to dry at any point during the procedure. Slides were treated with an exogenous peroxidase‐blocking solution (2% H_2_O_2_ in PBST) for 20 min, rinsed, and then treated with 1% bovine serum albumin solution. Slides were then exposed for 30 min to a ready‐to‐use 1:12,000 polyclonal rabbit anti‐human thyroglobulin primary antibody in liquid form (Dako, Denmark A/S). After several washes in PBS+ Tween (PBST), slides were treated with Anti‐Rabbit EnVision System horseradish peroxidase‐labeled polymer secondary antibody (Dako, Denmark A/S). NovaRed substrate kit for peroxidase (Vector) was applied for 5 min for visualization. Slides were then washed in DI water, taken through a series of ethanol washes, mounted in Permount, and coverslipped.

### 2.7 Hematoxylin and Eosin staining

De‐paraffined horizontal sections from at least two individuals of each treatment were stained using Gill's hematoxylin and eosin (H&E) using standard staining methods (Gill et al., 1974). De‐paraffinized and rehydrated slides were taken stepwise through a series of 1‐min ethanol washes. Gill's hematoxylin #2 was applied to the slides for 4 min, and then rinsed followed by treatment with eosin for 3 min. Slides were then dehydrated in an ethanol series and coverslipped in Permount.

### 2.8 Single‐cell RNA‐Seq

Zebrafish of the Nadia stock (Wilson et al., 2014) were raised under standard conditions (Westerfield, 1993). Animals were genotyped for a sex‐linked marker comprised of a CviQI (New England Biolabs) restriction enzyme site present in the Z chromosome but not in the W chromosome using primers F: CCGCGTTTATATCCTGGTAA and R: GTTGACCCAACTGGACTCTG and conditions of 6 min at 94°C and 45 cycles of 25 s at 94°C, 25 s at 61°C, 30s at 72°C, followed by 10 min at 72°C (Wilson et al., 2014). At 30 dpf, fish were euthanized in ice water, their gonads were dissected, and gonad cells were dissociated as in Wang et al. (2017) and Covassin et al. (2006). Cells were filtered through a 40‐micron strainer to form a single‐cell suspension and counted (Bio‐RAD TC20 Automated Cell Counter). The University of Oregon Genomics & Cell Characterization Core Facility constructed libraries with Chromium v2 chemistry (10x Genomics) and sequenced these libraries on an Illumina NextSeq 500 (Farnsworth et al., 2020). Sequences were aligned to GRCZ11 annotation version 92 (Zerbino et al., 2018) with Cell Ranger v. 2.0.1 (Zheng et al., 2017) and then analyzed with Seurat v. 2.20 using the standard pipeline (Butler et al., 2018) to identify clusters of cells with similar transcriptomes. For each identified cluster, the analysis pipeline outputs genes that are highly overexpressed with respect to all other clusters in the dataset, as well as the fraction of cells in each cluster expressing each gene and their statistical support. This information identified the most highly differentially expressed genes and the fraction of cells that express each gene in each cluster. From this gene list, transcriptomic cell types were annotated by querying gene expression information in ZFIN (Zebrafish Information Network, http://zfin.org/action/marker/search). Subsequently, hematopoietic and pancreas clusters were excluded, and gonad cells were re‐clustered. Data that support the findings of this study are available from the corresponding author upon reasonable request. All sequence data from this study have been archived at the Sequence Read Archive (www.ncbi.nlm.nih.gov/sra) under the accession number PRJNA504448.

## 3 RESULTS

### 3.1 The SLC5A gene family includes separate NIS clades and SGLT clades that are conserved across bony vertebrates

**Figure 1:**
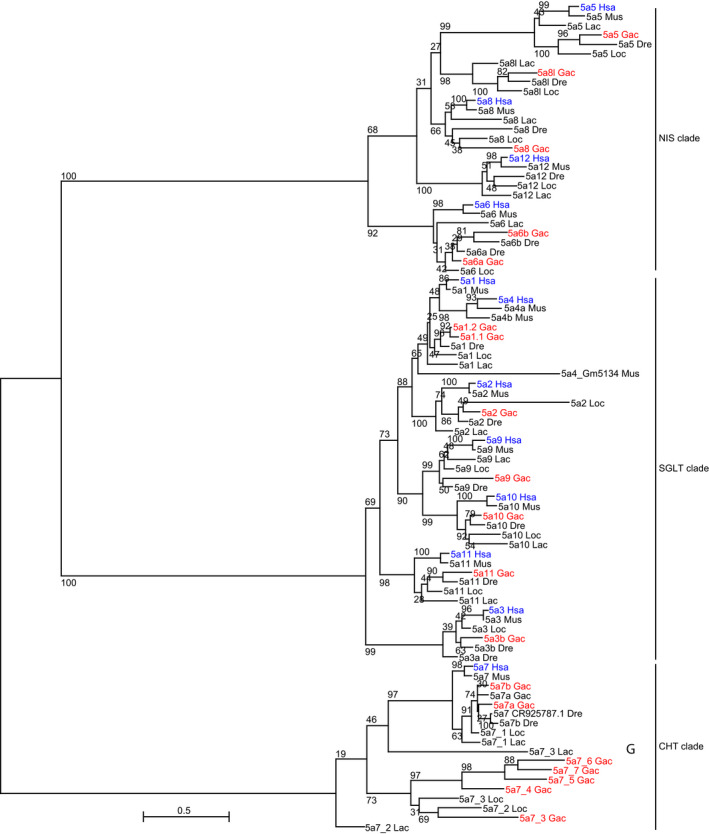
Maximum‐likelihood phylogenetic tree of Slc5a aligned amino acid sequences in representative vertebrate taxa. Phylogenetic analysis used a LG + G + F substitution model. A maximum‐likelihood tree inferred using PhyML (see Section 2) rooted here on the *CHT* clade. Numbers at nodes indicate bootstrap support (among 100 replicates). Protein names omit ”Slc”. Human and stickleback proteins are highlighted in blue and red, respectively. Results identified three main clades: the NIS (sodium–iodide symporter) clade at the top, the SGLT (sodium–glucose cotransporter) clade in the middle, and the *slc5a7* clade. Dre, *Danio rerio*; GAC, *Gasterosteus aculeatus;* Hsa, *Homo sapiens*; Loc, *Lepisosteus oculatus*; Mus, *Mus musculus*

We identified and aligned homologs of the *Slc5a5* sodium/iodide symporter from stickleback, zebrafish, gar, coelacanth, mouse, and human genomes (Figure 1, Figures [Link], [Link]). Phylogenetic analysis revealed an ingroup with two main clades: (1) the NIS clade, containing genes encoding proteins related to the sodium–iodide symporter (NIS), including *slc5a5, slc5a6, slc5a8, slc5a8l,* and *slc5a12*; and (2) the SGLT clade, containing genes encoding sodium–glucose transporters (SGLT), including *slc5a1, slc5a2, slc5a3, slc5a4, slc5a9, slc5a10,* and *slc5a11*. The outgroup clade contained only SLC5A7, a high‐affinity choline transporter (alias CHT1, Okuda et al., 2002) (Figure 1). These three clades encompass 13 groups of orthologous genes. The human *SLC5A1* and *SLC5A4* genes are just 100 kb apart on Hsa22, at a location where nonmammalian vertebrates have a single gene called *slc5a1*. This genomic arrangement and their sister group relationship in the tree (Figure 1) suggest an origin of *SLC5A1* and *SLC5A4* by tandem duplication in the mammalian lineage. This duplication likely took place after the divergence of therian mammals from prototherians, as this arrangement is noted in eutherians and marsupial genomes such as that of the gray short‐tailed opossum (Ensembl genes ENSMODG00000009915 and ENSMODG00000010004, respectively). Interestingly, prototherian genomes such as platypus (*Ornithorhynchus anatinus*) and Australian echidna (*Tachyglossus aculeatus*) suggest an independent duplication in the region specific to that lineage (see platypus Ensembl genes ENSOANG00000003573 and ENSOANG00000002165, and node 30,944,061 of gene tree ENSGT00940000155844). In summary, the ”*slc5a1”* gene in nontherian vertebrates is temporally equidistant from both *SLC1A1* and *SLC1A4*. Because 12 of 13 orthologous groups (all except Slc5a4) have members in both ray‐finned (e.g., gar, stickleback, and zebrafish) and lobe‐finned (coelacanth, mouse, and human) lineages, we conclude that at least those 12 orthologous groups were present in the last common ancestor of all bony vertebrates.

The NIS clade experienced gene losses and gains during evolution. Stickleback, like other investigated percomorph fishes, lacks an ortholog of *SLC5A12*. In contrast, the sister clade to percomorphs (Holocentriformes, which includes soldierfish), as well as zebrafish and more basally diverging teleosts, and lobe‐finned fish, including humans, have an *SLC5A12* ortholog (Ensembl gene tree ENSGT00940000159545), showing that this gene was present in their last common ancestor. Reciprocally, *slc5a8l* orthologs are present in the ray‐finned fish lineage as well as nontetrapod sarcopterygians (coelacanth and West African lungfish genomes) but absent from syntenic regions in available tetrapod genomes (e.g., *Xenopus* frog, turtle, chicken, opossum, and human), showing that the last common ancestor of all bony vertebrates had a copy of *slc5a8l*. Stickleback and other teleosts have two co‐orthologs of human *SLC5A6*, called *slc5a6a* and *slc5a6b*. The position and topology of these two lineages on the tree suggest that these duplicates arose in the teleost genome duplication event (TGD) (Amores et al., 1998; Braasch et al., 2016; Jaillon et al., 2004; Postlethwait et al., 1998), even though the topology of the tree was not exactly as predicted by a TGD origin. The SGLT clade has teleost duplicates of *SLC5A1,* and the SLC5A7 clade has several co‐orthologs of *SLC5A7* in stickleback.

### 3.2 Understanding the evolutionary trajectory of NIS‐clade genes

Conserved syntenies can reveal gene histories and help identify orthologs, whereas trees can sometimes mislead due to reciprocal lineage‐specific gene loss after genome duplication, especially independent gene losses in ray‐finned and lobe‐finned lineages after two rounds in the vertebrate genome duplications (VGD) (Dehal & Boore, 2005) and the TGD. Reciprocal ohnolog loss in two branches in a phylogenetic tree presents each as the other's closest sequence neighbor even though they are not orthologs. To overcome this problem, we analyzed genomic neighborhoods surrounding genes in the *slc5a5* clade.

**Figure 2:**
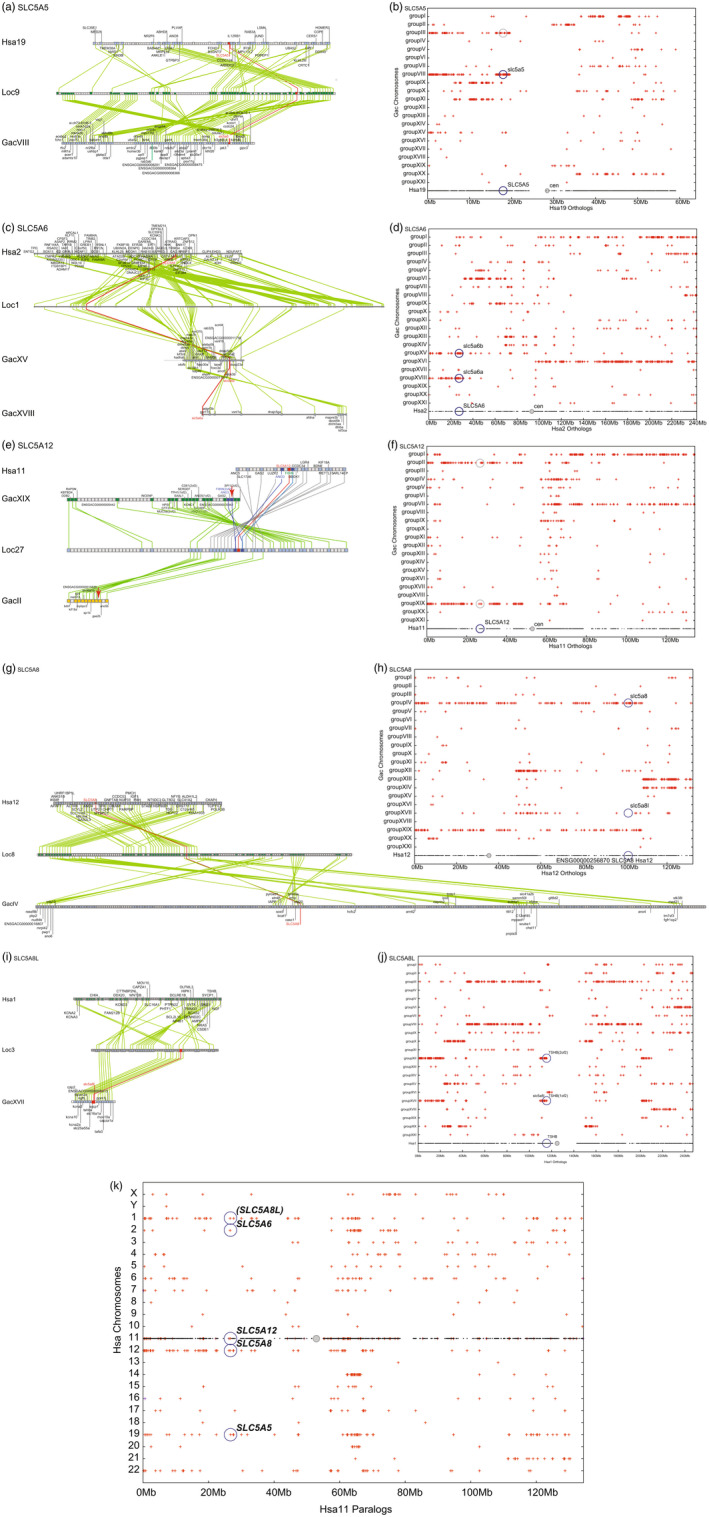
Conserved synteny analysis of *slc5a* gene family members in the NIS clade. (a, c, e, g, i) Ortholog comparison plots for human (Hsa, top), gar (Loc, middle), and stickleback (Gac, bottom). Red lines link orthologous *slc5a* family members. (b, d, f, h, j) Dot‐plot analyses showing a human chromosome on the horizontal axis with the location of its *SLC5A* gene marked; stickleback orthologs and close paralogs of human genes are displayed directly above the location of the human gene on a row indicating the chromosome on which the stickleback gene resides. For example, “b” shows the *SLC5A5* gene on human chromosome 19 and indicates its ortholog on stickleback linkage group GacVIII. Gene order in the horizontal dimension is that of the human chromosome. Chromosome regions that appear in duplicate due to the teleost genome duplication event are evident. (k) Dot plot of Hsa2 paralogs on other human chromosomes showing the location of NIS‐clade genes in paralogons. (l) Dot plot of Hsa22 paralogs on other human chromosomes showing the location of SGLT‐clade genes in paralogons

*SLC5A5* on human chromosome 19 (Hsa19) occupies a chromosome segment conserved on spotted gar chromosome Loc9 and stickleback linkage group GacVIII (Figure 2a). We examined a dot plot (Catchen et al., 2009) showing the location of stickleback orthologs and paralogs of genes on Hsa19 plotted on their stickleback chromosome in the order they appear on Hsa19 (Figure 2b). The stickleback orthologs of genes on the short (left) arm of human chromosome 19 (Hsa19p) were usually present on GacVIII and GacIII or on GacIX and GacXI (Figure 2b), which thus represent duplicated segments arising from the TGD. The stickleback *slc5a5* gene is on GacVIII, and its TGD duplicated copy would be expected to be on GacIII, but no *slc5a‐*related gene was identified at this location (Figure 2b). We conclude that: (1) conserved syntenies support the identity of stickleback *slc5a5* (ENSGACG00000008677) as the ortholog of human *SLC5A5,* and (2) the co‐ortholog of stickleback *slc5a5* was lost after the TGD.

The *SLC5A6* (*SMVT*, sodium‐dependent multivitamin transporter) gene is located in a region with syntenies conserved between human and gar (Figure 2c). Stickleback has two chromosome regions with conserved syntenies to the gar and human chromosome segments containing *slc5a6* and *SLC5A6,* with *slc5a6b* (ENSGACG00000011683) on GacXV and *slc5a6a* (ENSGACG00000004185) on GacXVIII (Figure 2c). The dot‐plot analysis (Figure 2d) shows that the telomeric 30 Mb of the short arm of human chromosome‐2 (Hsa2p) is present in two duplicated copies in stickleback, and that both have *SLC5A6‐*related genes. We conclude that the stickleback lineage retained both TGD co‐orthologs of *SLC5A6*.

*SLC5A12* (SMCT2, sodium‐coupled monocarboxylate transporter‐2) on Hsa11p is in a chromosome segment that contains 14 genes with orthologs mostly in the same order on gar chromosome Loc27, with *SLC5A12* lying between *ANO3* and *FIBIN* in both human and gar (Figure 2e). Phylogenetic analysis suggested that stickleback lacks a copy of *slc5a12* (Figure 1). To understand the mechanism underlying the *slc5a12* loss in stickleback after its lineage diverged from the gar lineage, we examined the corresponding regions of the stickleback genome. A copy of the gar *slc5a12‐*containing region appears on stickleback linkage group GacXIX, but no *slc5a*‐related gene lies between *ano3* and *fibin* (Figure 2e). A dot plot shows that almost the entire short arm of Hsa11 is duplicated on GacXIX and GacII (Figure 2f), and the region of GacII expected to contain an *slc5a12* paralog is also deleted (Figure 2e). We conclude that one TGD duplicate of *slc5a12* went missing from teleosts before the divergence of the zebrafish and stickleback lineage (Figure 1), and the copy in the other duplicated chromosome segment was lost by deletion in the percomorph lineage but remains in the genomes of extant, nonpercomorph teleosts such as zebrafish.

*SLC5A8* (SMCT1, sodium‐coupled monocarboxylate transporter‐1) on Hsa12 resides in a chromosome segment containing 18 genes with gar orthologs in the same order on Loc8, although some genes are missing in one or the other segment (Figure 2g). A dot‐plot analysis reveals the expected location of the TGD duplicate for *slc5a8* (ENSGACG00000019087) because the central portion of the long arm of Hsa12 is duplicated on stickleback chromosomes GacIV and GacXIX (Figure 2h), but no *slc5a* family gene occupies the expected position on GacXIX. We conclude that stickleback has a single copy of *slc5a8* on GacIV but that chromosome inversions have distorted gene orders.

The gene *slc5a8l* (ENSGACG00000005284) is missing from the human genome. The stickleback linkage group GacXVII has *slc5a8l* in the sequence of genes: *ngfb ‐‐ tspan2a ‐‐ slc25a55a ‐‐ tshba ‐‐ slc5a8l ‐‐ sycp1* (Figure 2I bottom). Coelacanth, which, like human, is a lobe‐finned fish, has an *SLC5A8L* gene (see tree, Figure 1) that resides on scaffold JH126587.1 in the sequence of genes *SYCP1‐‐SLC5A8L‐‐ SLC25A55A ‐‐TSPAN2‐‐NGF* as in stickleback, showing that *slc5a8l* existed before the divergence of ray‐finned and lobe‐finned fishes. Human chromosome Hsa1 has conserved syntenies with a region of gar chromosome Loc3 (Figure 2I), including the gene sequence *SYCP1‐‐TSPAN2—NGF,* but missing the central portion containing the three genes *zgc:92113, tshb (1 of 2),* and 404 *slc5a8l*. Gar chromosome Loc3 also preserves this gene order (Figure 2I middle). Human chromosome Hsa1 has conserved syntenies with a region of gar chromosome Loc3, including the gene sequence *NGF—TSPAN2‐‐TSHB‐‐ SYCP1,* but missing the central portion containing orthologs of the two genes *slc5a8l* and *slc25a55a* (Figure 2I, top). A dot‐plot analysis (Figure 2j) shows that stickleback has two copies of the portion of Hsa1 adjacent to orthologs of *tshb* and other genes flanking stickleback *slc5a8l*; one copy is on GacXVII, the location of *slc5a8l*, and the other copy is on GacXII, which lacks an *scl5a*‐related gene. Because both spotted gar in the ray‐finned lineage and coelacanth in the lobe‐finned lineage have an ortholog of *slc5a8l* (Figure 1)*,* we conclude that the last common ancestor of all bony vertebrates had *slc5a8l*, but a specific deletion of *slc5a8l* and *slc25a55a* orthologs removed *SLC5A8L* from the lobe‐finned lineage after the divergence of coelacanth and human lineages.

To investigate the origins of NIS‐clade genes, we plotted the paralogs of Hsa2 genes on other human chromosomes. Figure 2k shows that the portion of Hsa2 from about 25 Mb to about 67 Mb appears to be present in four paralogous copies in the human genome, as expected from two rounds of whole‐genome duplication before the vertebrate radiation. Three of these paralogons have NIS‐clade genes: Hsa2, *SLC5A6;* Hsa11, *SLC5a12*; and Hsa19, *SLC5a5*. The fourth paralogon appears to be broken, with the portion expected to contain *SLC5A8* (from about 25 to 32 Mb) on Hsa15 and the remainder of the paralogon (from about 32 to 67 Mb) on Hsa14. In contrast, *SLC5A8* is on Hsa12. Note that the right portion of Hsa2, from about 150 Mb to the telomere, is paralogous mostly to Hsa7, 12, and 17, so the origin of *SLC5A8* is unclear. Collectively, however, these data tentatively support the model that the members of the NIS clade that survive in the human genome likely arose in the two rounds of genome duplication at the base of vertebrate phylogeny.

### 3.3 Understanding the evolutionary trajectory of SGLT‐clade genes

Phylogenetic analysis shows that SLC5A1, SLC5A4, SLC5A2, SLC5A10, and SLC5A11 occupy the same clade; genes encoding these proteins reside on Hsa16, Hsa17, and Hsa22 (Figure 2l). The implicated portions of these three chromosomes were all part of a single chromosome in the last common ancestor of bony vertebrates that broke apart in the lineage leading to humans (Nakatani et al., 2007; Naruse et al., 2004; Postlethwait et al., 2004); thus, the most parsimonious explanation for their history is that these genes all arose by tandem duplications of a single gene on the single chromosome ancestor of Hsa16, 17, and 22, and that the breakup of that chromosome led to the tandem duplicates appearing on different chromosomes in the human lineage. Only two protein‐coding genes lie between *SLC5A1* and *SLC5A4* in the human genome as expected for tandem duplicates and *slc5a10* and *slc5a11* lie less than 1 Mb apart in the stickleback genome, again suggesting tandem duplication. Origins of the remaining SGLT subfamily genes – *SLC5A9* on Hsa1, *SLC5A3* on Hsa21, and the most basally diverging gene in the SLC5A clade, *SLC5a7* on Hsa2 – remain uncertain.

### 3.4 Thyroglobulin antigen appears in thyroid follicles at 8, 14, and 30 dpf

**Figure 3:**
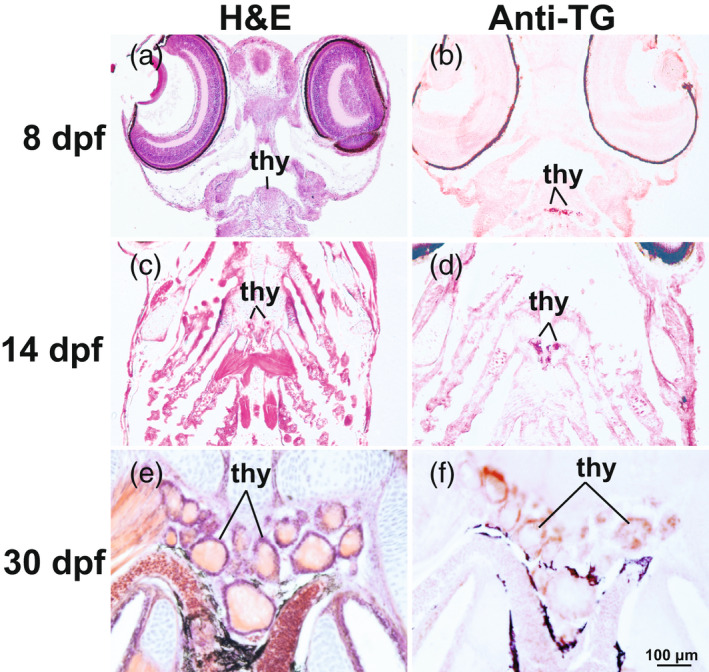
Colocalization of thyroid follicles and thyroglobulin protein at 8, 14, and 30 dpf in stickleback larvae. (a, c, e) Hematoxylin and eosin (H&E)‐stained horizontal sections of the pharynx showing thyroid follicles at 8–30 dpf. (b, d, f) Positive staining for thyroid antibody (anti‐TG [thyroglobulin]) is visible in and around the lumen of thyroid follicles at 8–30 dpf. Imaged at 400X. thy, thyroid follicles

Having identified stickleback orthologs of human *NIS‐*clade genes, including duplicates from the TGD and lineage‐specific losses of VGD paralogs, we examined the expression patterns of NIS‐clade genes to help understand the mechanisms of perchlorate toxicity. Initially, we investigated the development of the thyroid in histological sections of embryos and larvae. Analysis of H&E‐stained horizontal sections from stickleback larvae at 8 days postfertilization (dpf), about 1 day after hatching, revealed individual thyroid follicles distributed across the central pharynx near the branchial artery (Figure 3a,b). Thyroid follicles became larger and more defined by 14 dpf, and more follicles were present and dispersed medially in the pharynx along major branchial arteries including the ventral aorta (Figure 3c,d). By 30 dpf, mature thyroid follicles were large, bounded by epithelial cells, and contained a central colloid similar to human thyroid follicles. Thyroglobulin, the protein precursor of thyroid hormones, was already apparent in immuno‐stained developing thyroid follicles by 8 dpf and continued in juveniles (Figure 3b,d, f).

### 3.5 SLC5A5 paralogs are broadly expressed in developing stickleback

**Figure 4:**
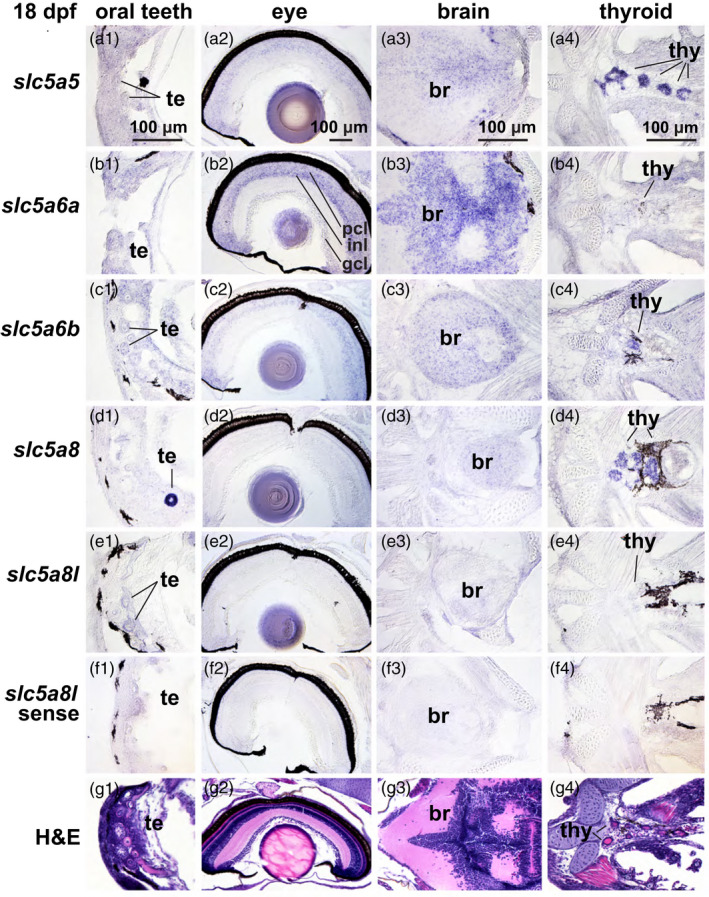
Expression patterns of *slc5a5* clade genes in the head of 18 dpf threespine stickleback. In situ hybridization of mRNA expression of (a) *slc5a5,* (b) *slc5a6a,* (c) *slc5a6b,* (d) *slc5a8,* (e) *slc5a8l,* and (f) *slc5a8l* sense strand as control, and (g) H&E‐stained histological sections showing teeth, eye, brain, and thyroid of larval stickleback. Imaged at 40X. br, brain; gcl, ganglion cell layer; inl, inner nuclear layer; pcl, photoreceptor cell layer; te, teeth; thy, thyroid follicles

**Figure 5:**
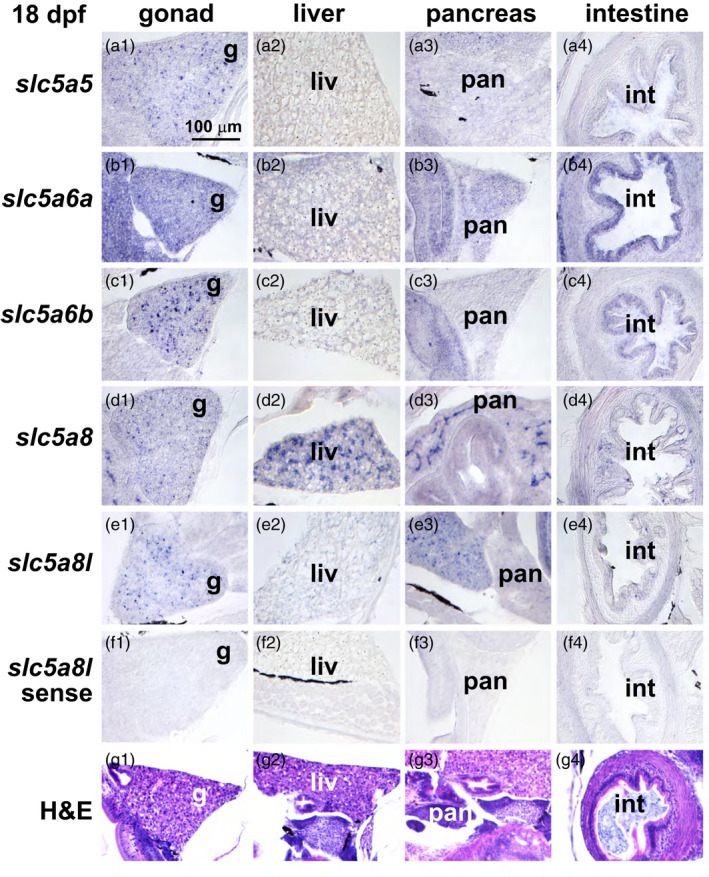
Expression patterns of *slc5a5* clade genes in the abdomen of 30 dpf threespine stickleback. (a) *slc5a5,* (b) *slc5a6a,* (c) *slc5a6b,* (d) *slc5a8,* (e) *slc5a8l*, and (f) *slc5a8l* sense strand as control, and (g) H&E‐stained histological sections showing gonad, liver, pancreas, and intestine imaged at 400X. g, gonad; int, intestine; liv, liver; pan, pancreas

In the 18dpf stickleback head, *slc5a5* expression appeared in the photoreceptor layer of the retina, medially in the brain, and strongest in the thyroid epithelium (Figure 4a). In the 18 dpf trunk, *slc5a5* was expressed in developing oocytes in the gonad and in the intestinal epithelium (Figure 5a). At 18 dpf, *slc5a6a* was expressed strongly in the photoreceptor cell layer, inner nuclear layer, and ganglion cell layer of the retina, medially in the brain, weakly in the thyroid (Figure 4b), in germ cells in the gonad, and strongly in the intestinal epithelium (Figure 5b). At this stage in the head, *slc5a6b* was expressed in the retina and medial brain, and rather strongly in the thyroid (Figure 4c); in the trunk, *slc5a6b* was expressed strongly in germ cells of the gonad and in the intestinal epithelium (Figure 5c). At 18dpf in the head, *slc5a8* was distinctive in its strong expression in the teeth and in the thyroid (Figure 4d), while in the trunk, it showed strong expression in the liver and pancreas (Figure 5d). The 18 dpf expression of *slc5a8l* in the head showed some expression in the teeth (Figure 4e) and in germ cells of the gonad and pancreas (Figure 5e).

**Figure 6:**
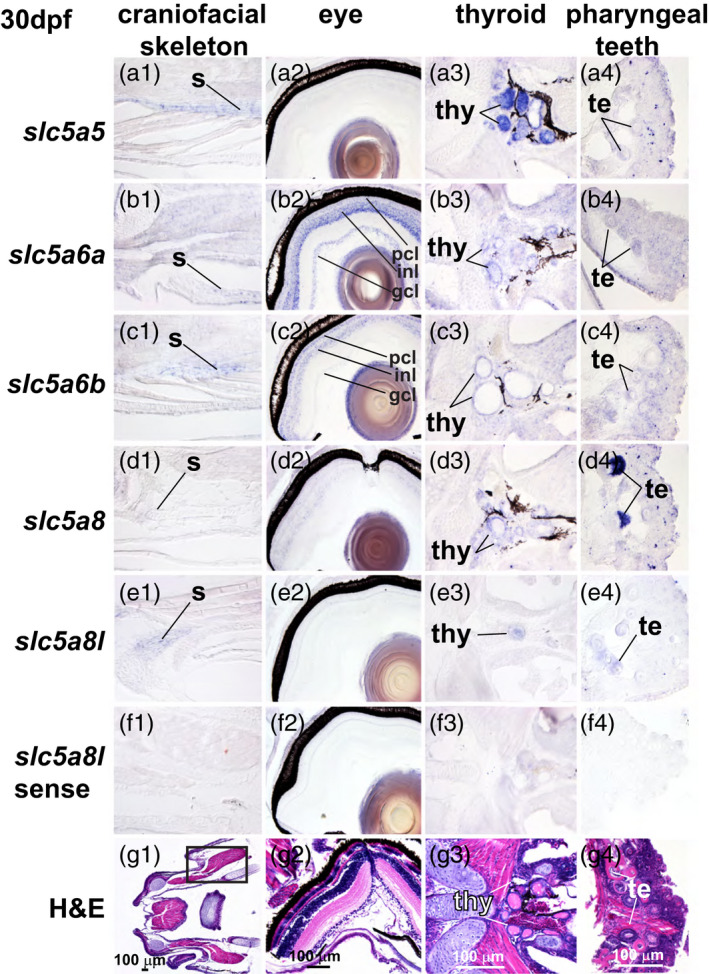
Expression patterns of *slc5a5* clade genes in the head of 30 dpf threespine stickleback. (a) *slc5a5,* (b) *slc5a6a,* (c) *slc5a6b,* (d) *slc5a8,* (e) *slc5a8l*, and (f) *slc5a8l* sense strand as control, and (g) H&E‐stained histological sections shown in the craniofacial skeleton, eye, thyroid, and pharyngeal teeth. Imaged at 400X. gcl, ganglion cell layer; inl, inner nuclear layer; pcl, photoreceptor cell layer; te, teeth; thy, thyroid follicles; s, skeletal tissue

**Figure 7:**
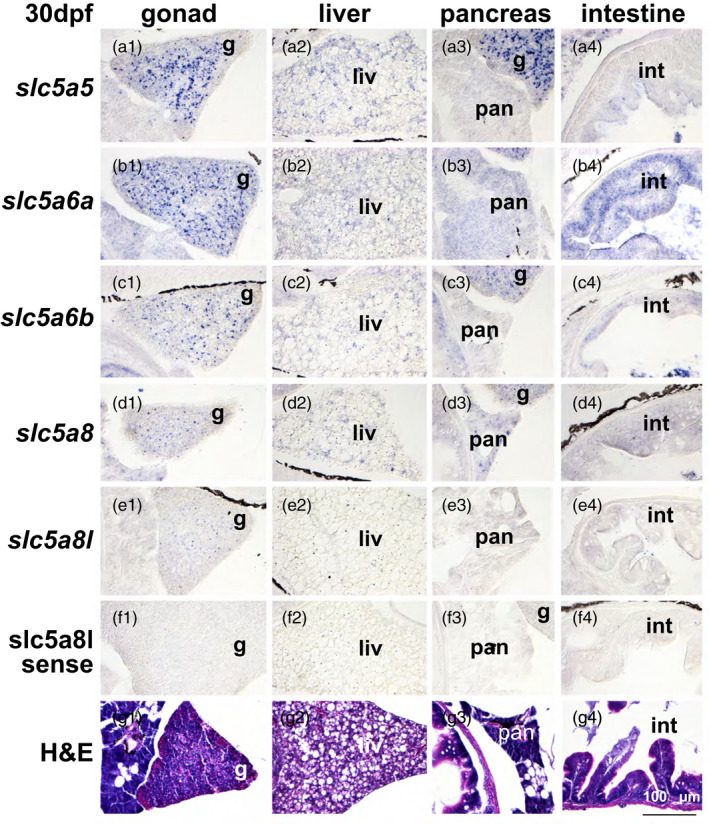
Expression of *slc5a5* clade genes in the abdomen of 30 dpf threespine stickleback. (a) *slc5a5,* (b) *slc5a6a,* (c) *slc5a6b,* (d) *slc5a8,* (e) *slc5a8l*, and (f) *slc5a8l* sense strand as control, and (g) H&E‐stained histological sections. Imaged at 400X. g, gonad; int, intestine; liv, liver; pan, pancreas

**Figure 8:**
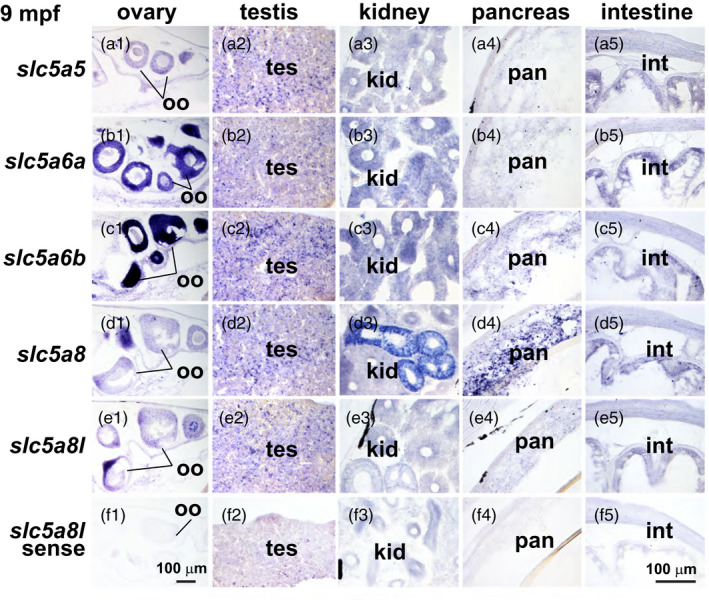
Expression of *slc5a5* clade genes in the abdomen of 9 mpf threespine stickleback. (a) *slc5a5,* (b) *slc5a6a,* (c) *slc5a6b,* (d) *slc5a8,* (e) *slc5a8l*, and (f) *slc5a8l* sense strand as control, and (g) H&E‐stained histological sections. Imaged at 400X. int, intestine; kid, kidney; oo, oocyte; pan, pancreas; tes, testis

In the 30 dpf stickleback head, *slc5a5* was expressed in the developing craniofacial skeleton and strongly in the thyroid follicles (Figure 6a), in the gonad in germ cells, and weakly in the liver and intestinal epithelium (Figure 7a). At 30 dpf, *slc5a6a* was distinctive among the paralogs in its strong expression in the retina, especially in the inner nuclear layer, but it was also expressed in the thyroid epithelium, germ cells in the gonad, and the intestinal epithelium (Figures 6b and 7b). The *slc5a6b* paralog was expressed in the craniofacial skeleton in a pattern similar to *slc5a5* as well as in the retina, but with a pattern different from *slc5a6a*. Specifically, *slc5a6b* was expressed more strongly in the photoreceptor cell layer relative to the inner nuclear layer than was *slc5a6a*. Additionally, *slc5a6b* was expressed less strongly in the ganglion cell layer relative to the other layers than was *slc5a6a* (Figure 6c). In the 30 dpf trunk, *slc5a6b* was expressed in the gonad and in the intestinal epithelium (Figure 7c). At 30 dpf in the head, *slc5a8* was expressed in thyroid follicles and, distinctively, in the teeth, as it was at 18 dpf (Figure 6d). In the abdomen at 30 dpf, *slc5a8* was expressed in the gonad, liver, and pancreas (Figure 7d). The head of 30 dpf stickleback expressed *slc8a8l* in skeletal elements, and weakly in thyroid follicles and teeth (Figure 6e). In the abdomen, a few gonad cells expressed *slc8a8l* (Figure 8e).

In 9‐month postfertilization stickleback (9 mpf), *slc5a6a* and *slc5a6b* were expressed stronger in the oocytes relative to other organs than the other paralogs, although all paralogs appeared to be expressed in gonads at some level (Figure 8a1‐f1). All of the paralogs appeared to be expressed in germ cells in the testes, which had not yet begun to develop mature sperm (Figure 8b2‐e2). The relative expression of *slc5a8* was stronger in the kidney and pancreas compared to the other paralogs (Figure 8a3‐e3 and a4‐e4).

Using in situ hybridization to compare expression across genes can be challenging, but comparing the relative expression of individual genes across various organs in the same sections is internally controlled. Expression of *slc5a8* was especially high in teeth relative to other organs, and this pattern was true to a lesser extent for *slc5a8l* (Figures 4d1, e1 and 6d4, e4). Craniofacial skeletal elements appeared to express nearly all the NIS‐clade genes rather weakly compared to other organs for each gene (Figure 6a1, b1, c1, d1, e1). Specific layers in the retina expressed *slc5a6a* and *slc5a6b*, but at different relative levels in different retinal layers. These two *slc5a6* ohnologs were also expressed in the brain, suggesting that they share functions involved in neuronal development or function in the retina and in the brain. The relative expression of *slc5a5* and *slc5a8* in the thyroid epithelium was high compared to their other expression domains. Gonads appeared to express all five NIS‐clade genes at 18 dpf, apparently in oocytes, although at 30 dpf, *slc5a8* expression appeared to down‐regulate. Liver and pancreas appeared to express *slc5a8* more strongly relative to its expression in other organs in contrast to the other NIS‐clade paralogs, especially at 18 dpf (Figure 5d3). The intestine appeared to express *slc5a6a* more strongly relative to its signal in other organs.

### 3.6 Expression of NIS‐clade genes at the single‐cell level in gonads

**Figure 9:**
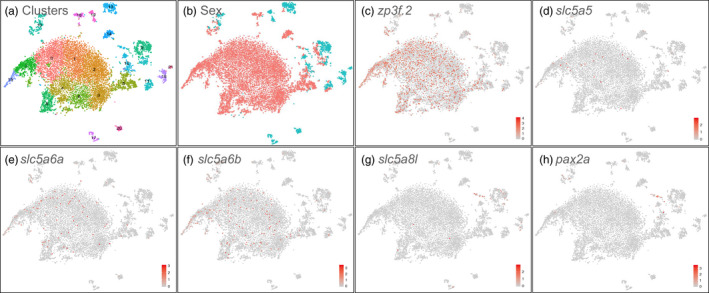
Single‐cell RNA‐seq analysis of 30 dpf zebrafish gonads. (a) tSNE plot with clusters numbered. Each dot represents a cell; (b) Cells from genetically female (red) and male (blue) gonads; (c) *zp3f.2* eggshell protein gene expression, darker red indicates higher expression level according to the legend in the corner; (d) *slc5a5* expression in maturing oocytes; (e) *slc5a6a* expression in a few immature ZW germ cells and in many maturing oocytes; (f) *slc5a6b* expression in a few immature ZW germ cells and in many maturing oocytes; (g) *slc5a8l* expression primarily in somatic cells of the gonad; and (h) *pax2a* expression in the same cluster as *slc5a8l*. The scale bar gives the natural log‐transformed feature counts for each cell divided by the total counts for that cell, multiplied by the scale factor (a value of 1 is added to each count before the calculation to avoid taking the log of 0)

To verify the identity of cell types expressing NIS‐clade genes in fish gonads, we used single‐cell transcriptomics (scRNA‐seq). Because this methodology requires well‐annotated genomes with extensive documentation of gene expression patterns, we turned to zebrafish so that analysis could exploit gene expression information in the Zebrafish Information Network database (http://zfin.org/). We dissected gonads from 30 dpf zebrafish juveniles and performed single‐cell RNA‐sequencing (scRNA‐seq) experiments. We utilized the Nadia strain, which has, like stickleback, a chromosomal sex determination system, and identified genetic sex by PCR (Wilson et al., 2014). Some of the transcriptomic cell clusters were sex specific (clusters 0, 14, 26, 17, 18, and 21) while others had mixtures of genetic male and female cells (clusters 9, 10, 11, and 12) (Figure 9a,b). To identify germ cells, we interrogated the data for clusters containing cells expressing germ‐cell markers, including *ddx4* and the eggshell protein gene *zp3f.2,* which labeled clusters 0 and 10, and identified these clusters as germ cells (Figure 9c). Among NIS‐clade genes, *slc5a5*, *slc5a6a*, and *slc5a6b* were all expressed in germ cells (Figure 9d–f), while *slc5a8l* was expressed in an uncharacterized set of cells of the gonadal soma in cluster 12 that was also the main group of cells expressing *pax2a* (Figure 9g,h), which in chicken forms gonad supporting cell lineages (Sertoli/pregranulosa cells) (Estermann et al., 2020). Expression of *slc5a8* and *slc5a12* was not detected in these scRNA‐seq experiments. We conclude that three of the five NIS‐clade genes that were expressed in stickleback juvenile gonads (Figures 5 and 7) were also expressed in oocytes in juvenile zebrafish and a fourth was expressed in a specific small group of gonadal soma cells known to be important for gonadal development in other vertebrates (Figure 9).

## 4 DISCUSSION

Perchlorate is a pervasive contaminant in many parts of the world. For example, it was detected in 83 of 84 serum and plasma samples of United States residents (Oldi & Kannan, 2009) and in the urine of all 2820 U.S. residents tested (Blount et al., 2002). Studies on various mammals in both the wild and the laboratory found that perchlorate exposure affects litter size, increases stillborn frequency, and reduces ossification in offspring, although the magnitude of changes was usually small (Stoker et al., 2006; Thuett et al., 2002; York et al., 2001; York et al., 2003). Aquatic vertebrates that are chronically exposed to perchlorate have altered body size and shape (Bernhardt et al., 2011), increased angiogenesis and thyroid follicle proliferation (Furin, von Hippel, Cresko, et al., 2015b), disrupted immune function (Capps et al., 2004), liver pathology (Minicozzi et al., 2019), altered development of dermal bone (Furin, von Hippel, Postlethwait, et al., 2015a), and changed gonadal development and sex determination (Bernardt & von Hippel, 2008; Bernhardt et al., 2006; Petersen et al., 2015, 2016; Sharma & Patiño, 2013).

A major action of perchlorate is to bind the sodium–iodide symporter Slc5a5 and inhibit its ability to sequester iodide into the thyroid, a process that is essential for the production of thyroid hormones (Lawrence et al., 2000; Leung et al., 2010). An open question is whether the effects of perchlorate on reproduction are mediated solely by its effects on the thyroid, or whether perchlorate might have direct effects on other organs. The gonad/paralog hypothesis predicts that perchlorate acts directly on other organs in addition to the thyroid by interacting with SLC5a5’s closely related paralogs. Thus, we first identified genes in the vertebrate genome closely related to Slc5a5, verified their evolutionary relationships to human proteins, and then examined expression profiles of *slc5a5‐*related paralogs in stickleback, a model organism for ecotoxicology (Katsiadaki et al., 2007).

### 4.1 Evolutionary history of the SLC5A family

The results of phylogenetic analyses showed that the *slc5a* family has two well‐supported, reciprocally monophyletic clades, one that contains sodium–glucose cotransporters, the SGLT clade, and one that contains paralogs more closely related to Slc5a5, the NIS clade (Figure S1). Within the SGLT clade, we identified one case of differential resolution of TGD‐derived duplicates among teleost lineages: *Slc5a3* has likely retained both co‐orthologs in the zebrafish lineage, but just one in the stickleback lineage, which could potentially make stickleback a more tractable model for future studies of *Slc5a3* function. For the remainder of the Discussion, however, we focus on the NIS clade, which is evolutionarily most closely related to *Slc5a5*.

Analysis of phylogenetic relationships coupled to the analysis of conserved syntenies showed that the last common ancestor of teleost fish and humans about 450 million years ago had five members of the NIS clade (*Slc5a5*, *Slc5a6*, *Slc5a8*, *Slc5a8l,* and *Slc5a12*). Analysis of dot plots for paralogs of genes on human chromosome 2 generally support the hypothesis that these genes evolved during two rounds of vertebrate genome duplication events, VGD1 and VGD2. Furthermore, the immediate syntenic regions in the human genome for *SLC5A8L* (the individual gene was lost in tetrapods)*, SLC5A8,* and SLC5A12 all share predicted homology with reconstructed proto‐vertebrate chromosome 11 (Nakatani et al., 2021), although the SLC5A5 region corresponds to different proto‐vertebrate chromosomes (Pvcs 13 and 14). This is not completely consistent with both VGD events giving rise to the genes above, but it is possible that ancient translocation of a small region including SLC5A5 explains the discrepancy.

Future structural analysis should investigate whether and which particular regions of *Slc5a5* orthologs make them uniquely susceptible (as a subclade) to binding iodine‐like contaminants (e.g., perchlorate). If so, a useful avenue of research would investigate which regions of the proteins bind strongly to iodine‐like contaminants, and whether other NIS‐clade paralogs are sufficiently divergent in these regions to reduce their likelihood as perchlorate targets. The evolutionary relationships of the *Slc5a5* subclade to *Slc5a8, Slc5a8l*, and *Slc5a12* subclades (its closest paralog relatives) were not well resolved in our analysis, but each paralog group within the NIS clade showed strong evidence of monophyly. We therefore consider each subclade a meaningful evolutionary unit and discuss important patterns for several of these groups within the context of our study.

After the divergence of ray‐finned and lobe‐finned vertebrates, several events occurred that distinguish the NIS‐clade content of stickleback and human. The *slc5a8l* gene was retained in the ray‐finned lineage of bony vertebrates but was lost in lobe‐finned bony vertebrates after the divergence of the coelacanth lineage from the tetrapod lineage. Our expression pattern data did not identify any organs or stages in stickleback that were expressing *slc5a8l* to the exclusion of other *slc5a* family genes. Thus, *slc5a8l* might have been redundant in the tetrapod lineage, or modularly associated with traits and/or functions lost in that lineage, and its loss may have had little phenotypic consequence for natural selection. If this supposition is correct, then the remaining question is what evolutionary force, adaptive or otherwise, might have maintained this gene in ray‐finned vertebrates?

NIS‐clade gene loss also occurred in the ray‐finned lineage. Although many teleosts retained an ortholog of *slc5a12* (including zebrafish and other otophysans such as Mexican tetra (*Astyanax mexicanus*) and catfish (*Silurus glanis*), as well as more basally diverging teleosts such as herring (*Clupea harengus*), pike (*Esox lucius*), and arowana (*Osteoglossum bicirrhosum*)), none of the very large group of percomorph fish appears to possess this gene (see gene name “ENSGT00940000159545” in Ensembl). In zebrafish, *slc5a12* is expressed in the pronephros in 5dpf larvae (Thisse & Thisse, 2004). The loss of *slc5a12* from the genome of stickleback and other percomorph fish may relate to redundancy of the kidney expression domain with expression of *slc5a8* in the kidney that we observed. Although the absence of *slc5a12* rules out its role in the toxicological effects of perchlorate on percomorphs such as stickleback, future studies of *slc5a12* in nonpercomorph models such as zebrafish and other vertebrate lineages are important, particularly regarding kidney development and function.

In addition to lineage‐specific gene loss, additional NIS‐clade genes appear in the ray‐finned lineage. Stickleback and other teleosts have two copies of *slc5a6*. Analysis of phylogenies suggested, and conserved syntenies showed, that these two genes arose in the TGD (Amores et al., 1998; Jaillon et al., 2004; Postlethwait et al., 1998). Furthermore, the expression of these two genes distinctively in the retina with weaker expression in the thyroid and teeth is consistent with inherited redundancy in expression domains. The relative expression in different layers of the retina, however, differs between the two ohnologs as predicted by the duplication, degeneration, and complementation hypothesis (Force et al., 1999), and that difference might account for their retention in duplicate copy in the 350 million years since the TGD. Similar expression domain divergence between *slc5a6a* and *slc5a6b* may exist for tissues more directly linked to perchlorate toxicity as well. For example, our in situ hybridization results in stickleback suggest stronger expression of *slc5a6b* than *slc5a6a* in 18 dpf thyroid follicles, and in germ cells. Precise quantification of expression changes for these paralogs (in these spatiotemporal contexts) upon experimental exposure to perchlorate should be a focus of future work. Likewise, an expanded comparative analysis of conserved noncoding elements (CNEs) in *slc5a6* regions among vertebrates could reveal candidate cis‐regulatory regions driving ancestrally shared vs. derived expression domains.

### 4.2 Thyroid follicle distribution, morphology, and function in juvenile stickleback

Stickleback thyroid follicles develop in the central pharynx starting shortly after hatching at 8 dpf. Thyroid follicles cluster near the ventral aorta and each follicle resembles thyroid follicles of other teleosts and humans, with an epithelium surrounding a colloid (Wendl et al., 2002). The organization of thyroid follicles in stickleback and other teleosts, however, differs from follicle organization in mammals: rather than a unitary thyroid organ, teleost thyroid follicles are loosely distributed ventral to the pharynx (Wendl et al., 2002). Our demonstration that anti‐human thyroglobulin identifies thyroglobulin protein in thyroid follicular cells suggests that thyroid follicles in stickleback and human share the functions of iodide uptake and thyroglobulin production (De Felice, 2004; Wendl et al., 2007). The close apposition of thyroid follicles and blood vessels in the stickleback thorax may accommodate signaling from the vascular epithelium that is important for the differentiation of thyroid follicles (Alt et al., 2006). Results showed that at 14 dpf, thyroglobulin protein expression has become more robust, and by 30 dpf, thyroid follicles have obtained their characteristic adult shape, structure, and function of thyroglobulin production as thyroid hormones begin to affect the metamorphosis from larva to juvenile (Power et al., 2001).

### 4.3 NIS‐clade gene expression patterns

We examined the spatial distribution of NIS‐clade transcripts in developing stickleback. If NIS‐clade paralogs are not expressed in organs disrupted by perchlorate treatments, then they do not provide a plausible mechanism for the developmental disruptions caused by perchlorate (Furin, von Hippel, Cresko, et al., 2015b, Petersen et al., 2016).

Results revealed that *slc5a5* transcript is expressed in 18 and 30 dpf stickleback in numerous nonthyroidal organs, including oral teeth, eye, brain, gonad, and thyroid (Figure S3). The expression of *slc5a5* in these organs is compatible with the hypothesis that they play a role in the development or physiology of cells in addition to the thyroidal epithelium. Radioiodide studies in humans have likewise demonstrated *slc5a5* expression in organs in addition to the thyroid, including prostate, mammary gland, lung, heart, salivary gland, stomach, and placenta (Portulano et al., 2014). Craniofacial regions where we detected *slc5a5* expression in stickleback have cellular contributions from, or physical proximity to, migrating cells from the neural crest (Nagoshi et al., 2009). Neural crest cells are multipotent, forming such diverse structures as neurons, glial cells, myofibroblasts, pigment cells, adipocytes, chondrocytes, osteocytes, and connective tissue (Nagoshi et al., 2009), suggesting broad implications for perchlorate interference with expression of *slc5a5* directly in these cell types.

While Slc5a5 resides in the basal membrane of mammalian thyrocytes and transports iodide from the blood into the cell, Slc5a8 lies in the apical membrane of thyrocytes and transports iodide into the cell from the colloid (Rodriguez et al., 2002). Our in situ hybridization results show that *slc5a5* and *slc5a8* are both strongly expressed in stickleback thyrocytes, consistent with conserved functions. Slc5A8 also transports monocarboxylates and short‐chain fatty acids, and, in the kidney, is a high‐affinity sodium–lactate cotransporter (Gopal et al., 2004; Thangaraju et al., 2006). Our results showed that stickleback kidneys also strongly express *slc5a8*. Slc5a12 serves as a low‐affinity sodium–lactate cotransporter in the kidney (Gopal et al., 2004; Thangaraju et al., 2006), but as our phylogeny shows, stickleback lacks an *slc5a12* gene, suggesting that *slc5a8* in percomorph fishes may perform the nephric roles of *Slc5a12* in mammals. In stickleback, *slc5a8* was also strongly expressed in developing teeth, suggesting that its role in transport of monocarboxylates and short‐chain fatty acids by a sodium‐coupled mechanism that occurs during enamel production is shared with mammals, even though teleosts do not make enamel (Braasch et al., 2016; Kawasaki, 2009; Kawasaki & Amemiya, 2014). Endocrine disruptors such as Bisphenol A alter *Slc5a8* expression in mammalian teeth (Jedeon et al., 2016) and they might do so in stickleback as well. In stickleback, *slc5a8* is expressed in the kidney, as it is in zebrafish (Plata et al., 2007).

In our study, *slc5a6a* and *slc5a6b* were prominently expressed in the eye and brain (Figure S3). NIS‐clade genes in humans are important in brain and retinal pigment epithelial cells for the uptake of myo‐inositol, a solute important for cell volume homeostasis (Berry et al., 2003; Boles & Krämer, 2004). In humans, SLC5A6 is also an active transporter of biotin, an essential nutrient readily absorbed by cells in the retina (Kansara et al., 2006). Although *slc5a6a* and *slc5a6b* have broadly overlapping expression patterns in stickleback, each has its own distinctive characteristics – their relative strength of expression in different layers of the retina and in the intestine differs. This finding suggests that the maintenance of the two ohnologs might be due to subfunctionalization, the reciprocal loss of ancestral genetic regulatory elements (Force et al., 1999). The stickleback expression profile for *slc5a8l* closely resembles that for its zebrafish ortholog and for the zebrafish *slc5a12* gene (Plata et al., 2007). The redundant expression patterns of these two genes in zebrafish may help explain how *slc5a12* might have disappeared in the lineage of stickleback and other percomorphs, and reciprocally, the *slc5a8l* gene was lost in tetrapods.

Our findings support a broad pattern of NIS‐clade gene expression in the stickleback head, thorax, and trunk. A central pattern to emerge from these data is that the craniofacial bone and cartilage, retina, and several visceral organs including liver, pancreas, and intestines express NIS‐clade genes during larval and juvenile development and during adulthood. The broad expression of these paralogs in nonthyroidal organs also raises the possibility that these tissues may be more susceptible to disruption by chemicals that are disruptors of NIS‐clade genes. The substrate flexibility of these transporters might further contribute to ease of disruption of NIS‐clade proteins; for example, Slc5a5 transports a variety of molecules, including thiocyanate, nitrate, and tetraflouraborate, as well as at least eight other compounds, including perchlorate (Eskandari et al., 1997) (Figure S3).

All five stickleback NIS‐clade genes were expressed in gonads of both sexes. In undifferentiated gonads in larvae and juveniles, and in young adult testes, NIS‐clade genes were expressed in puncta that appeared to be immature germ cells. The mammalian *SLC5A5* gene is also expressed in fetal testis in mouse, rat, and human and immunohistochemistry revealed that SLC5A5 protein was localized in germ cells and Leydig cells, but not in Sertoli cells (Russo et al., 2011). Cancer of the testis is the most frequent cancer in young men, and nearly all germ cell testicular tumors, but not Leydig cell tumors, express *SLC5A5* (Micali et al., 2013), making radioiodine a possible therapy. We found that stickleback testes express not only *slc5a5* but also *slc5a6a*, *slc5a6b*, slc5a8, and *sl5a8l*. We also found transcripts encoding NIS‐clade paralogs in the ooplasm of stickleback oocytes, where these symporters likely aid in the uptake of free fatty acids and other essential teleost yolk nutrients (Patiño & Sullivan, 2002). Our single‐cell transcriptomic (scRNA‐seq) experiments in zebrafish verified that *slc5a5*, *slc5a6a*, and *slc5a6b* are expressed in immature oocytes and immature germ cells of 30 dpf juvenile zebrafish, and that *slc5a8l* is expressed in a specific type of gonadal soma cell that is important for gonadal function in chickens (Estermann et al., 2020). Expression of *slc5a8* and *slc5a12* was not detected in zebrafish gonads at this stage, but the lack of *slc5a8* expression in scRNA‐seq data (while present in corresponding in situ experiments) could reflect insufficient sequencing depth for lowly expressed genes. These results from zebrafish confirm the conserved expression of several NIS‐clade genes in teleost gonads. Our data suggest that these versatile multisubstrate transporters should be investigated for possible disruption by Slc5a5 competitors.

## 5 CONCLUSIONS

Results reported here outline the evolutionary history of *SLC5A‐*family genes, thereby connecting them authentically to their human orthologs (Figure S1). Furthermore, we identify expression domains conserved in threespine stickleback, zebrafish, and human that suggest similar functions conserved across vertebrates. The novel expression domains reported here, especially expression in developing and mature gonads, suggest that stickleback is a good model in which to test questions about the roles of members of this gene family in reproductive development and pathologies, including testicular cancers. Our findings also raise the question of whether compounds in the environment such as perchlorate may have previously unknown and unsuspected effects on reproductive development. Our previous work provides evidence that perchlorate may affect development in a nonthyroidally mediated mechanism, and our evidence for widespread expression domains of multiple NIS‐clade paralogs reveals a possible mechanism to support this conclusion. Future work should investigate specific effects of perchlorate on regulation of NIS‐clade genes in stickleback and other model vertebrates.

## CONFLICT OF INTEREST

The authors declare no conflict of interest.

## Supporting information


Fig. S1

Fig. S2

Fig. S3

Fig. S4

Fig. S5

## Data Availability

Data that support the findings of this study are available from the corresponding author upon reasonable request. All sequence data from this study have been archived at the Sequence Read Archive (www.ncbi.nlm.nih.gov/sra) under the accession number PRJNA504448.
