# Supplementary figures and images for: Evolution and developmental expression of the sodium–iodide symporter ( NIS , slc5a5) gene family: Implications for perchlorate toxicology
Source: Evol Appl. 2022 Jul 7;15(7):1079–98. doi: 10.1111/eva.13424 (PMC9309457; doi:10.1111/eva.13424)

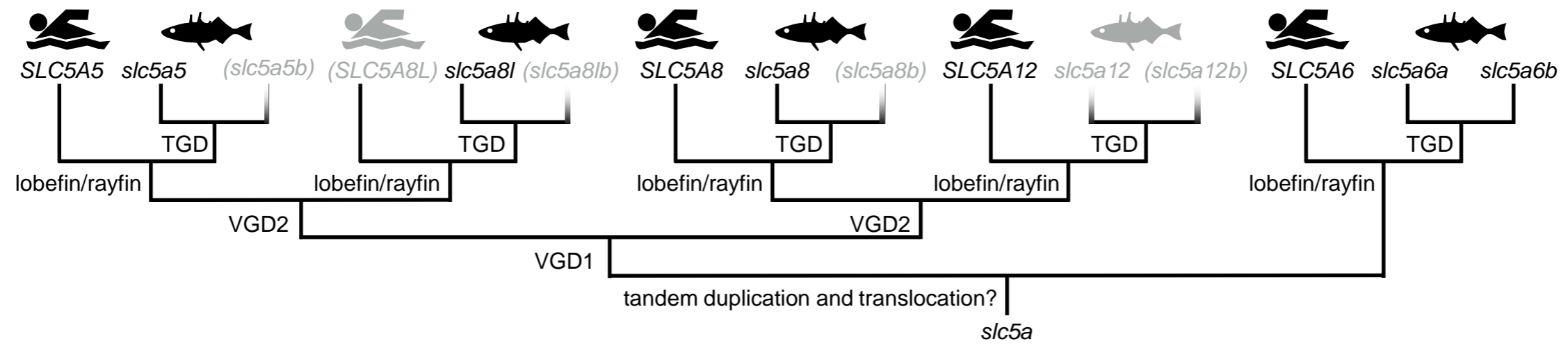

Supplement: Supplementary file 1 — Fig. S1 [file EVA-15-1079-s002.pdf]
