# Supplementary material for: Evolution and developmental expression of the sodium–iodide symporter ( NIS , slc5a5) gene family: Implications for perchlorate toxicology
Source: Evol Appl. 2022 Jul 7;15(7):1079–98. doi: 10.1111/eva.13424 (PMC9309457; doi:10.1111/eva.13424)
Supplement: Supplementary file 2 — Fig. S2 [file EVA-15-1079-s005.pdf]

SLC5s_MAFFTaln_trimmed_key

Name in tree	Accession #
**slc5a1.1 Gac**	ENSGACT00000022832
**slc5a1.2 Gac**	ENSGACP00000022808
**slc5a1 Dre**	ENSDART00000080449
**slc5a1 Hsa**	ENST00000266088
**slc5a1 Loc**	ENSLOCT00000001617
**slc5a1 Lac**	ENSLACP00000019875
**slc5a1 Mmu**	ENSMUST00000011178
**slc5a2 Dre**	ENSDART00000171841
**slc5a2 Gac**	ENSGACT00000007722
**slc5a2 Hsa**	ENST00000330498
**slc5a2 Loc**	ENSLOCT00000001774
**slc5a2 Lac**	ENSLACP00000008286
**slc5a2 Mmu**	ENSMUST00000118169
**slc5a3b Dre**	ENSDART00000115265
**slc5a3a Dre**	ENSDARP00000009841
**slc5a3 Gac**	ENSGACT00000004882
**slc5a3 Hsa**	ENST00000381151
**slc5a3 Loc**	ENSLOCT00000022495
**slc5a3 Mmu**	ENSMUST00000113975
**slc5a4 Hsa**	ENSP00000266086
**slc5a4b Mmu**	ENSMUSP00000113582
**slc5a4a Mmu**	ENSMUSP00000020450
**slc5a4_Gm5134 Mmu**	ENSMUSP00000097172
**slc5a5 Gac**	ENSGACP00000011465
**slc5a5 Hsa**	ENST00000222248
**slc5a5 Mmu**	ENSMUST00000000809
**slc5a5 Loc**	ENSLOCT00000003298
**slc5a5 Lac**	ENSLACP00000016154
**slc5a5 Dre**	ENSDART00000114699
**slc5a6b Dre**	ENSDARP00000062951
**slc5a6a Dre**	ENSDARP00000114963
**slc5a6a Gac**	ENSGACP00000005542
**slc5a6b Gac**	ENSGACP00000015462
**slc5a6 Has**	ENSP00000310208
**slc5a6Loc**	ENSLOCP00000019578
**slc5a6 Lac**	ENSLACP00000005159
**slc5a6 Mmu**	ENSMUSP00000006817
**slc5a8 Gac**	ENSGACP00000025245
**slc5a8 Dre**	ENSDARP00000137715
**slc5a8 Has**	ENSP00000445340
**slc5a8 Loc**	ENSLOCP00000018706
**slc5a8 Lac**	ENSLACP00000013594
**slc5a8 Mmu**	ENSMUSP00000020255
**slc5a8l Gac**	ENSGACP00000007041
**slc5a8l Dre**	ENSDARP00000030637
**slc5a8l Loc**	ENSLOCP00000013048
**slc5a8l Lac**	ENSLACP00000021102
**slc5a9 Gac**	ENSGACT00000002792
**slc5a9 Hsa**	ENST00000533824
**slc5a9 Mmu**	ENSMUST00000102721
**slc5a9 Loc**	ENSLOCT00000011825
**slc5a9 Lac**	ENSLACP00000018324
**slc5a9 Dre**	ENSDART00000024433
**slc5a10 Dre**	ENSDART00000112160
**slc5a10 Gac**	ENSGACT00000022165
**slc5a10 Has**	ENST00000395647
**slc5a10 Loc**	ENSLOCT00000007024
**slc5a10 Lac**	ENSLACP00000012659
**slc5a10 Mmu**	ENSMUST00000051552
**slc5a11 Dre**	ENSDARP00000048217
**slc5a11 Gac**	ENSGACP00000015597
**slc5a11 Has**	ENSP00000289932
**slc5a11 Loc**	ENSLOCP00000006440
**slc5a11 Lac**	ENSLACP00000017115
**slc5a11 Mmu**	ENSMUSP00000127977
**slc5a12 Dre**	ENSDARP00000013866
**slc5a12 Has**	ENSP00000379326
**slc5a12 Loc**	ENSLOCP00000007853
**slc5a12 Lac**	ENSLACP00000016588
**slc5a12 Mmu**	ENSMUSP00000047340
**slc5a7_2 Gac**	ENSGACP00000009017
**slc5a7_1 Gac**	ENSGACP00000020280
**slc5a7_3 Gac**	ENSGACP00000024877
**slc5a7_4 Gac**	ENSGACP00000024446
**slc5a7_5 Gac**	ENSGACP00000023294
**slc5a7_6 Gac**	ENSGACP00000015216
**slc5a7_7 Gac**	ENSGACP00000015161
**slc5a7b Dre**	ENSDARP00000134712
**slc5a7_CR925787.1 Dr**	ENSDARP00000131632
**slc5a7a Dre**	ENSDARP00000117033
**slc5a7**	ENSP00000264047
**slc5a7_1 Loc**	ENSLOCT00000012772
**slc5a7_2 Loc**	ENSLOCP00000009026
**slc5a7_3 Loc**	ENSLOCP00000009044
**slc5a7_1 Lac**	ENSLACP00000006231
**slc5a7_2 Lac**	ENSLACP00000002252
**slc5a7_3 Lac**	ENSLACP00000010766
**slc5a7 Mmu**	ENSMUST00000095712
