# Supplementary material for: Evolution and developmental expression of the sodium–iodide symporter ( NIS , slc5a5) gene family: Implications for perchlorate toxicology
Source: Evol Appl. 2022 Jul 7;15(7):1079–98. doi: 10.1111/eva.13424 (PMC9309457; doi:10.1111/eva.13424)
Supplement: Supplementary file 3 — Fig. S3 [file EVA-15-1079-s004.docx]

| Gene name | Expression Domains in Developing Stickleback | Substrate transported by orthologs to this gene in humans and other vertebrates |
| --- | --- | --- |
| *slc5a5* | - Photoreceptor of retina - Thyroid - Oocytes - Intestinal epithelium - Craniofacial skeleton | - Iodide - Sodium |
| *slc5a6a* | - Retina - Brain - Intestine - Germ cell/Gonad - Liver - Pancreas | - Sodium - Biotin - Panthothenate - Lipoate |
| *slc5a6b* | - Retina - Brain - Thyroid - Germ cell/Gonad - Intestinal epithelium | - Sodium - Biotin - Panthothenate - Lipoate |
| *slc5a8l* | - Teeth - Germ cell - Pancreas - Gonad | - Sodium - Iodide - Monocarboxylates - Short chain fatty acids |
| *slc5a8* | - Teeth - Thyroid - Liver - Pancreas | - Sodium - Iodide - Monocarboxylates - Short chain fatty acids |

Table 1. SLCA5 gene family *slc5a5, slc5a6, slc5a8, slc5a8l* expression domains in stickleback fish and known transport substrates of the orthologs of these genes humans and other vertebrates. See Discussion section for interpretation and references.
